# Effects of Trehalase on the Gene Expression of the Reproductive Regulation Pathway Network and Triglyceride Metabolism in *Nilaparvata lugens* (Stål)

**DOI:** 10.3390/insects16070725

**Published:** 2025-07-16

**Authors:** Bin Tang, Yuxin Ge, Yongkang Liu, Liwen Guan, Ye Han, Yang Zhu, Gao Hu, Yan Wu

**Affiliations:** 1 College of Life and Environmental Sciences, Hangzhou Normal University, Hangzhou 311121, China; geyuxin107@163.com (Y.G.); hznu_yongkang@163.com (Y.L.); guanliwen1010@163.com (L.G.); 19011285720@163.com (Y.H.); 2023112010008@stu.hznu.edu.cn (Y.Z.); 2 Guizhou Key Laboratory of Agricultural Biosecurity, Guiyang University, Guiyang 550005, China; hugao@njau.edu.cn; 3 State Key Laboratory of Agricultural and Forestry Biosecurity, College of Plant Protection, Nanjing Agricultural University, Nanjing 210095, China

**Keywords:** trehalase, reproduction, RNA interference, validamycin, *Nilaparvata lugens*

## Abstract

The high reproductive performance of *Nilaparvata lugens* (Stål) (Hemiptera: Delphacidae) is one of the key factors contributing to its serious damage. Studies have demonstrated that trehalose hydrolysis, catalyzed by trehalase (TRE), plays an important role in the reproductive regulation of *N. lugens*. However, it remains unclear how TRE regulates the reproduction of *N. lugens*. To address this question, the current study was designed to investigate the effects of TRE on the reproductive regulatory network of *N. lugens*. Specifically, RNA interference (RNAi) was conducted by injecting double-stranded RNA (dsRNA) targeting the TRE genes, and a trehalase inhibitor, validamycin, was used to suppress TRE activity. Subsequently, the relative expression levels of key genes involved in the reproductive regulatory pathway, as well as the triglyceride content in the fat body and ovary, were determined. The results indicated that both dsTREs and validamycin treatment decreased the relative expression of Vitellogenin (Vg) without affecting the expression of its receptor, *VgR*. However, their impact on the reproductive regulatory network differed: dsTREs injection did not affect the relative expression of *JHAMT* and *Met* in the juvenile hormone signaling pathway, but resulted in the upregulation of *USP* and *TOR*, and inhibition of *InR2* and *S6K*. In contrast, validamycin injection inhibited the expression of *Met*, *USP*, *EcR*, *InR1*, *TOR*, and *S6K*. Both dsTREs and validamycin inhibited the expression of *Fas*, but only validamycin decreased the ovarian triglyceride content. These findings suggest that the inhibition of *TRE* leads to alterations in reproductive regulatory signals, thereby reducing the reproductive capacity of *N. lugens*.

## Simple Summary

Trehalase (TRE) plays a critical role in insect survival and reproduction. The inhibition of TRE in the *Nilaparvata lugens* has been shown to impair reproductive capacity, yet the underlying molecular mechanisms have remained elusive. By inhibiting TRE activity (RNAi and validamycin), we discovered that TRE disruption fundamentally impacts the insect’s reproductive regulatory network and triglyceride metabolism pathways. This study provides crucial molecular insights into how trehalose metabolism governs insect reproduction. Importantly, it solidly validates TRE as a highly promising target for developing novel, mechanism-based strategies to control pest insects.

## 1. Introduction

*Nilaparvata lugens* (Stål) (Hemiptera: Delphacidae) exhibits multiple harmful behavioral traits that negatively impact rice production, contributing to its status as the most significant biotic threat to rice yield [1,2,3]. Currently, the predominant method for managing insect pests is the foliar application of insecticides [4]. However, this approach is not effective in the long-term control of pest populations and, when misused, can lead to the development of insecticide resistance through metabolic changes or target site mutations [5,6,7]. Given the severe consequences of *N. lugens* infestations on agricultural productivity, there is an urgent need to develop novel, environmentally friendly, sustainable, and efficient pest management strategies. Among the proposed strategies, suppressing the reproductive capacity of *N. lugens* emerges as a critical approach.

The metabolism of trehalose, a major circulating sugar during the growth of insect oocytes, has a key physiological role in insect reproduction [8]. Therefore, it has the potential to serve as a target for insecticide action. Trehalases (TREs) are pivotal enzymes involved in insect development and reproduction, including soluble (TRE1) and membrane-bound (TRE2) types [9,10,11]. TRE1 has two coding genes, *TRE1-1* and *TRE1-2*, while TRE2 has only one coding gene [12,13,14,15,16,17]. Previous studies showed that both TRE1 and TRE2 regulate development, molting, and emergence in insect larvae through the chitin pathway [15]. For example, the single interference of *TRE1-1*, *TRE1-2*, and *TRE2* genes all led to an imbalance in trehalose supply, thus increasing larval mortality in *N. lugens* [15]. Validamycin is a competitive natural inhibitor of TRE [18]. As a structural analog of trehalose, validamycin exhibits potent inhibitory activity against trehalase in pest insects [19,20,21,22,23,24], further supporting its potential as a target for insecticide action. This is in agreement with our laboratory’s previous results showing the significant inhibition of TRE activity following validamycin injection into *N. lugens* [16]. Studies have shown that the inhibition of TRE activity by validamycin can result in a significant decrease in ATP levels, leading to slow oocyte development [25]. This further confirms that functional TRE is essential for energy production in the ovary, and that its impairment affects reproduction.

Vitellogenin (Vg) is a key nutrient required for ovary development, and it is synthesized as a macromolecule mainly in the fat body and then transported to the mature oocyte via endocytosis, which is mediated by the vitellogenin receptor (*VgR*). This process, known as vitellogenesis, is a central event in insect reproduction [17,26]. Deficiencies in Vg synthesis and transport can directly lead to maternal sterility or the reduced hatchability of offspring [27]. It has been shown that vitellogenesis is mainly regulated by hormone signaling pathways [28]. Among them, the JH signaling pathway is involved in the regulation of vitellogenesis and oocyte maturation in some female insects and has an impact on other aspects of reproduction [29,30,31,32,33,34,35,36]. Moreover, 20-hydroxyecdysone (20E), another major regulatory hormone of female reproduction in some Hymenoptera, Lepidoptera, and all Diptera, is usually synthesized in the ovary [31,36,37]. The insulin-like peptide signaling pathway (IIS), also known as the insulin signaling pathway, plays a key role in various aspects of metabolism, development, and reproduction in insects [38,39]. Additionally, in vivo studies in *N. lugens* have demonstrated that the transcription factor Foxo can directly act on the exon to regulate *Vg* expression [40], indicating that the IIS can regulate insect reproduction independently of JH and 20E [41]. Furthermore, the IIS may crosstalk with the JH or 20E signaling pathways to indirectly affect insect reproduction [42]. The TOR signaling pathway, a key nutrient sensor, regulates physiological activities according to the organism’s nutritional status. After entering the cytoplasm through transmembrane receptors, amino acids bind to GTPases or Rag proteins, which results in the activation of the TOR pathway [43]. S6 kinase (S6K), phosphorylated and activated downstream of TOR, participates in the transcriptional and translational regulation of Vg [43,44]. Thus, the TOR signaling pathway directly regulates insect reproduction in response to nutrient availability. Furthermore, the TOR stimulates the secretion of insulin-like peptides in insects, thereby activating the IIS signaling pathway and indirectly influencing reproductive processes [43]. Therefore, four signaling pathways—JH, 20E, IIS, and TOR—constitute the reproduction regulatory network in insect reproduction [45,46,47,48].

Preliminary laboratory studies have shown that TRE plays an important role in the reproduction regulation of *N. lugens* [49]. However, it is not clear how TRE regulates the reproduction of *N. lugens*. Therefore, in this study, the TRE in *N. lugens* was inhibited in vivo at the mRNA and protein levels via dsTREs and validamycin injections, respectively, and the effects of TRE on the reproduction regulatory network were determined. The results of the study are conducive to a more comprehensive assessment of the ability of TRE as a pest control target and provide theoretical support for the development of new and safe biopesticides.

## 2. Materials and Methods

### 2.1. Insects

The *N. lugens* insects used in this study were obtained from local rice fields at the China National Rice Research Institute (Hangzhou, China). The institute was established in 2013. The rice variety used during the experiment was Taichung Native 1 (TN1), which was used to feed *N. lugens*. The environmental conditions of the artificial climate chamber were set as follows: temperature 27 ± 1 °C, relative humidity 65 ± 5%, and photoperiod 18L: 6D (Light: Dark). All experiments were performed under these same conditions to ensure consistency.

### 2.2. dsRNA Synthesis and Microinjection

Twenty adult *N. lugens* were randomly divided into 4 groups (five adults per group), which were then crushed in an EP tube and added to Trizol (Takara, Kyoto, Japan) for RNA extraction. After a series of operations, the obtained RNA was added to an appropriate amount of DEPC water and completely dissolved. Then, 1 μL of RNA solution was detected using the micro nucleic acid protein concentration analyzer Nanodrop2000 (Thermo Fisher, Waltham, MA, USA) to determine the RNA purity and concentration. RNA integrity was detected via 1% agarose gel electrophoresis. Using the extracted RNA as a template, cDNA was synthesized using the Prime Script RT reagent Kit with gDNA Eraser (Takara, Kyoto, Japan). The in vitro synthesis of dsRNA was performed using the T7 RiboMAXTM Express RNAi System (Promega, Madison, WI, USA), a dsRNA synthesis kit specifically designed for this purpose. Newly emerged long-winged females were selected for injection with the TransferMan 4r microinjector (Eppendorf, Hamburger, Germany). The dsRNA concentration was 4000 ng/μL, the validamycin concetration was 0.5 μg/μL, and each group was injected with 100 nL [15,16]. The dsTREs used in this study has successfully verified the inhibitory effect on *TRE1-1*, *TRE1-2*, and *TRE2* gene expression in earlier studies of our laboratory, with an inhibitory duration of 6 days [49]. The accession numbers and primer sequences of these three genes are presented in Table 1.

### 2.3. qRT-PCR

Five females were selected for RNA extraction and cDNA reverse transcription. The specific primers (Table 2) were synthesized, and the actin gene was used as an internal reference gene [27,45,46,50,51,52]. Subsequently, the synthesized cDNA was used as a template for qRT-PCR. The qRT-PCR was conducted with TB Green Premix Ex Taq™ (Takara, Kyoto, Japan). QRT-PCR was performed on a Bio-Rad CFX96TM real-time PCR detection system (Bio-Rad, Hercules, CA, USA) with the following reaction system: ddH_2_O 3.2 μL, TB Green Premix Ex Taq 5 μL, forward primer 0.4 μL, reverse primer 0.4 μL, and cDNA 1 μL. The specificity of the amplification reaction was judged by the amplification curve as well as the melting curve, and the relative expression of the genes was calculated according to the 2^−△△CT^ method [53].

### 2.4. Observation of Abdomen and Detection of Triglyceride Content

The injected female *N. lugens* was paired with untreated male *N. lugens* in a 1:1 ratio. The female *N. lugens* (*n* = 20, consistent with Section 2.2 cohorts) abdomen was observed and photographed at a magnification of 20×. After three days, the fat bodies and ovaries of these females were collected. The weight of each tissue sample was recorded using an electronic balance. Differences in the triglyceride determination methods were applied for fat body and ovary samples. Anhydrous ethanol was added to the fat body samples to form homogenates, while 1 × PBS was used in another set of preparations. And 10% homogenates were formed by grinding and cell crushing, respectively. After the reaction, both sets of samples were then processed with a triglyceride assay kit (Nanjing Jiancheng, Nanjing, China). The absorbance was measured at 510 nm using an enzyme-labeled microplate reader. The darker color produced during the reaction is proportional to the triglyceride content. Additionally, since ovaries are not high-fat samples, the protein content is also tested to normalize the triglyceride content. Protein was detected using the BCA protein detection kit (Beyotime, Shanghai, China). The triglyceride content in fat bodies was calculated as (Sample OD − Blank OD)/(Calibration OD − Blank OD) × Calibrator concentration (mmol/L). For ovaries, the triglyceride content was adjusted by dividing the above result by the protein concentration in the samples to be measured (gprot/L).

### 2.5. Data Analysis

Microsoft Excel 2019 was used to compile the data, GraphPad Prism 9.0.0 was used for graphing, and IBM SPSS Statistics 23.0 was used for statistical analysis. All data were presented as the means ± standard errors (SEs) of biological replicates, and Student’s *t* test was used to analyze the difference.

## 3. Results

### 3.1. Effects of Trehalase on Abdominal Volumes, Vg, and VgR Expression

**Figure 1:**
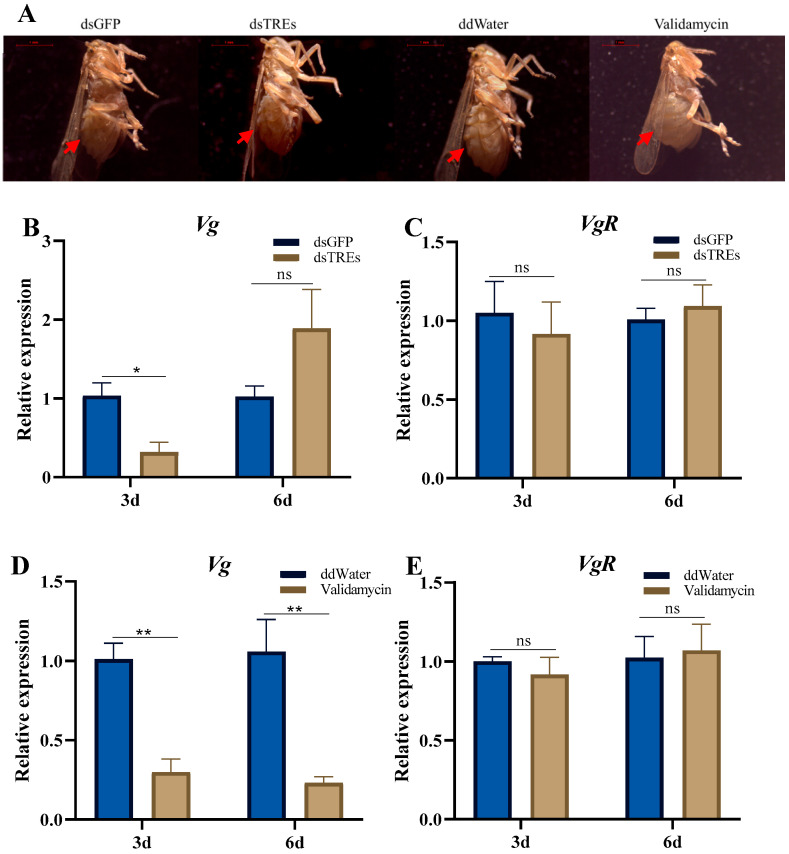
Comparison of abdominal volumes in *N. lugens* following injections (**A**) (Scale bar: 1 mm). The relative expression levels of *Vg* (**B**,**D**) and *VgR* (**C**,**E**) were detected on the third and sixth day after dsTREs injection and validamycin injection. Those injected with dsGFP and ddWater are the control group, and those injected with dsTREs and validamycin are the experimental group. (**A**) The shooting time was the third day after the injection. The magnification of the abdomen was 20×. The mRNA levels of *Vg* (**B**,**D**) and *VgR* (**C**,**E**) in *N. lugens* were detected on the third and sixth day after injection of dsTREs and validamycin, and the effects of trehalase on *Vg* and *VgR* expression were investigated. The data was shown as mean ± standard errors (n ≥ 3) and analyzed using Student’s *t* test. “**”, *p* < 0.01; “*”, *p* < 0.05; “ns”, *p* > 0.05.

The results showed that on the third day after the injection of dsGFP, dsTREs, and ddwater, the abdomens of *N. lugens* exhibited no significant differences. In contrast, those injected with validamycin exhibited abdominal enlargement, with the intersegmental membrane of the sternum fully distended (Figure 1A). The mRNA level of the *Vg* gene decreased significantly on the third day after dsTREs injection, but recovered by the sixth day, showing no significant difference compared to the control (Figure 1B). The mRNA level of *VgR*, on the other hand, remained comparable to that of the control group both on the third and sixth day after interference (Figure 1C). When *N. lugens* were injected with validamycin, the expression level of *Vg* in its body was extremely suppressed both on the third and sixth day (Figure 1D). However, similar to the results of dsTREs injection, the mRNA level of *VgR* did not change significantly compared with the control group, indicating that its expression was not affected by the suppression of TRE (Figure 1E).

### 3.2. Effects of Trehalase on JH and 20E Signaling Pathways

**Figure 2:**
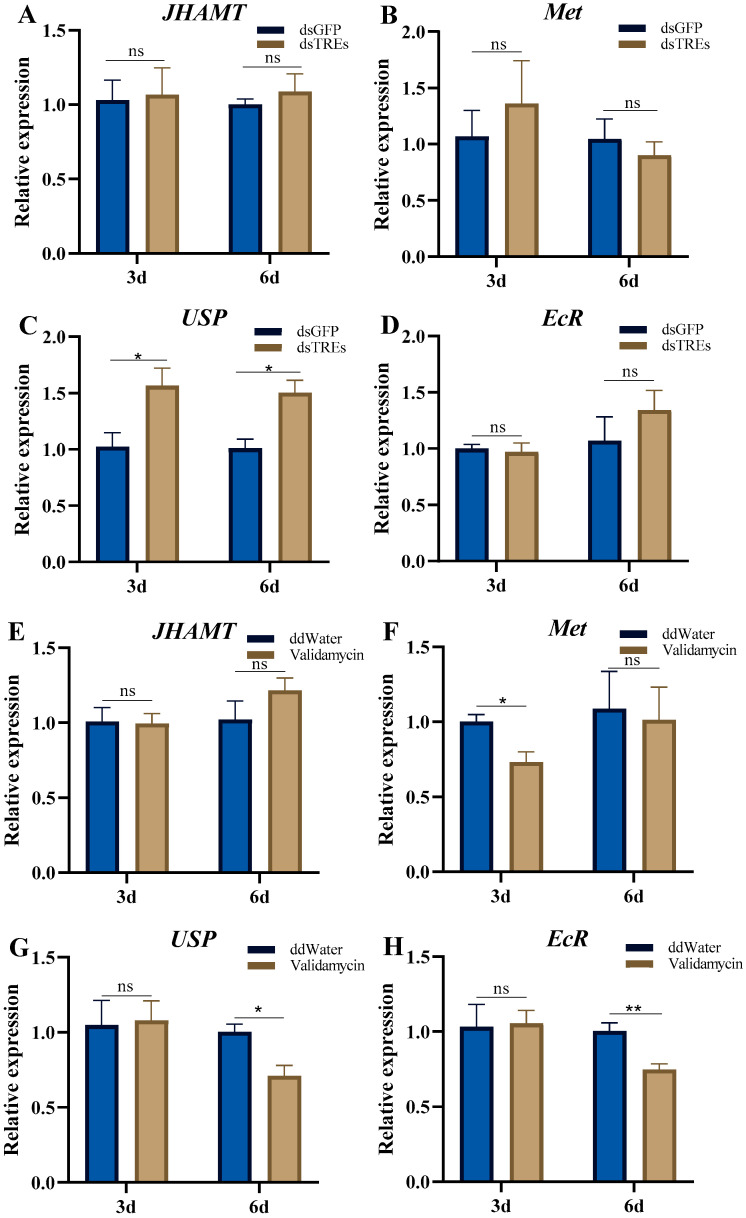
Effects of trehalase on JH and 20E signaling pathways. The relative expression of genes related to juvenile hormone signaling and ecdysone signaling on the third and sixth day after dsTREs and validamycin injection. The mRNA levels of *JHAMT* (**A**,**E**), *Met* (**B**,**F**), *USP* (**C**,**G**), and *EcR* (**D**,**H**) in *N. lugens* were detected on the third and sixth day after injection of dsTREs and validamycin, and the effects of trehalase on juvenile hormone signaling and ecdysone signaling expression were investigated. The data was shown as mean ± standard errors (n ≥ 3) and analyzed using Student’s *t* test. “**”, *p* < 0.01; “*”, *p* < 0.05; “ns”, *p* > 0.05.

The expression level of the 20E receptor *USP* significantly increased on the third and sixth day (Figure 2C). The expression level of another 20E receptor, *EcR,* was also not significantly different compared to the control group on the third day or sixth day after dsTREs injection (Figure 2D). Similarly, the mRNA levels of *JHAMT* were comparable to controls on the third and sixth day after validamycin injection, but *Met* expression was significantly downregulated on the third day after validamycin injection, suggesting that validamycin reduced signaling in the JH pathway (Figure 2E,F). The changing trends of *USP* and *EcR* levels after validamycin injection were similar. There was no significant difference on the third day, but both showed a significant decrease by the sixth day. This suggests that validamycin reduced signaling in the 20E pathway; however, its effect was delayed compared to that on the JH signaling pathway (Figure 2G,H).

### 3.3. Effects of Trehalase on Nutrient Signaling Pathways

**Figure 3:**
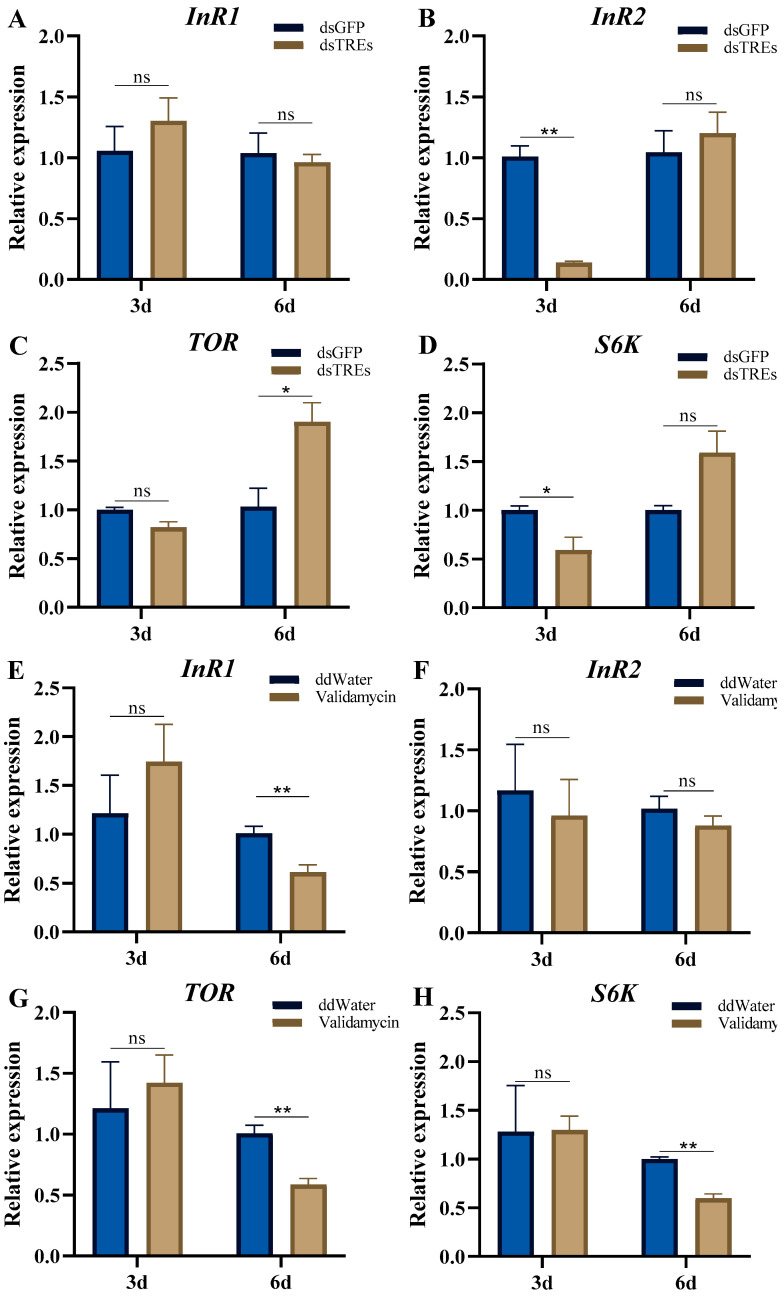
Effects of trehalase on nutrient signaling pathways. The relative expression of genes related to juvenile hormone signaling and ecdysone signaling on the third and sixth day after dsTREs and validamycin injection. The mRNA levels of *InR1* (**A**,**E**), *InR2* (**B**,**F**), *TOR* (**C**,**G**), and *S6K* (**D**,**H**) in *N. lugens* were detected on the third and sixth day after injection of dsTREs and validamycin, and the effects of trehalase on juvenile hormone signaling and ecdysone signaling expression were investigated. The data was shown as mean ± standard errors (*n* ≥ 3) and analyzed using Student’s *t* test. “**”, *p* < 0.01; “*”, *p* < 0.05; “ns”, *p* > 0.05.

Two insulin receptor genes, *InR1* and *InR2*, have been identified in *N. lugens* [50]. When the expression of *NlTRE1-1*, *NlTRE1-2*, and *NlTRE2* was simultaneously inhibited [49], the qRT-PCR results revealed no significant change in *InR1* expression on the third and sixth day compared with controls (Figure 3A,B). In contrast, *InR2* expression decreased significantly on the third and sixth day compared with controls (Figure 3A,B). *TOR* expression did not differ significantly from the control group on the third day after dsTREs injection, but increased significantly on the sixth day (Figure 3C). However, the specific indicator shown in Figure 3D exhibited a decrease on the third day and an increase after the sixth day compared to the dsGFP-injected group, although this difference was not statistically significant (Figure 3D).

When the TRE activity was inhibited by validamycin injection, *InR1* expression showed no significant change on the third day, but decreased highly significantly after the sixth day. *InR2* expression remained comparable to the control levels on both the third day and the sixth day of inhibition (Figure 3E,F). Similarly, the trends in the expression of key genes in the *TOR* signaling pathway were inconsistent with the results of dsTREs interference. On the third day after validamycin injection, *TOR* and *S6K* expression did not differ significantly from controls, but both were very significantly reduced on the sixth day (Figure 3G,H).

### 3.4. Effects of Trehalase on Lipid Metabolism

**Figure 4:**
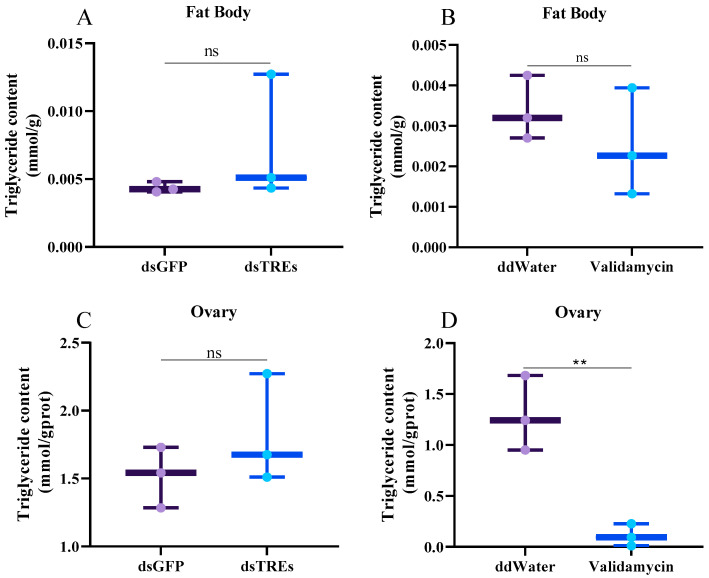
The triglyceride content in the fat body and ovary of *N. lugens* was measured on the third day following the injection of dsTREs and validamycin. The effect of trehalase on lipid metabolism was analyzed by measuring the content of triglyceride in fat body (**A**,**B**) and ovary (**C**,**D**) on the third day of injection. The data was shown as mean ± standard errors (*n* = 3) and analyzed using Student’s *t* test. “**”, *p* < 0.01; “ns”, *p* > 0.05.

The experimental results showed that three days after dsTREs injection, the content of triglycerides in both the fat body and ovaries did not differ significantly from that of the control group (Figure 4A,C). When injected with validamycin on the third day, the triglyceride content in the fat body of females remained unaffected, but the triglyceride content in the ovaries decreased very significantly, suggesting that the trehalase inhibitor prevented the accumulation of triglycerides in the ovaries (Figure 4B,D).

**Figure 5:**
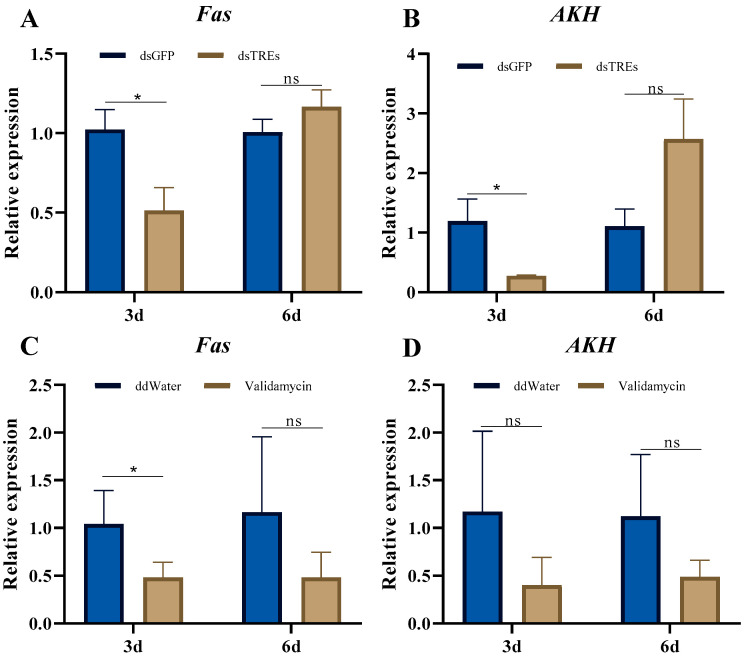
The relative expression of *Fas* (**A**,**C**) and *AKH* (**B**,**D**) of *N. lugens* on the third and sixth day after dsTREs injection and validamycin injection. The effect of TRE interference or activity inhibition on lipid metabolism of *N. lugens* was detected by qRT-PCR dsTREs. The data was shown as mean ± standard errors (*n* ≥ 3) and analyzed using Student’s *t* test. “*”, *p* < 0.05; “ns”, *p* > 0.05.

Using qRT-PCR, we further examined the effects of TRE interference or activity inhibition on lipid metabolism. The results revealed that the mRNA level of fatty acid synthase (*Fas*) decreased significantly three days after dsTREs injection, but recovered to control level by the sixth day (Figure 5A). The relative expression of Adipokinetic hormone (*AKH*), responsible for lipid mobilization, remained comparable to the control levels on both the third and sixth day (Figure 5B). After validamycin injection, the relative expression of *Fas* decreased substantially on both the third and sixth day, but this difference was statistically significant only on the third day (Figure 5C). Similarly, the relative expression of *AKH* decreased substantially on both the third and sixth day, although neither change was statistically significant (Figure 5D).

## 4. Discussion

Vitellogenin (Vg) plays a crucial role in the reproduction of *N. lugens*. *Vg* expression significantly increases after female emergence, and any disruption to this process results in infertility and abnormal oocyte development in *N. lugens* females [27]. The transporter receptor for *Vg*, *VgR*, is also highly expressed following emergence; interference with its function prevents Vg from accumulating in the ovary, causing decreased female fecundity [54]. Previous research on *Spodoptera frugiperda* indicated that the injection of trehalase inhibitors reduced *Vg* expression [17]. Consistent with these findings, our experimental results demonstrated that both dsTREs and validamycin led to the downregulation of *Vg* expression on the third day after injection (Figure 1A,C). Notably, *Vg* expression recovered later in the season after dsTREs injection (Figure 1A), while validamycin exhibited a more prolonged effect (Figure 1C), which correlated with egg production outcomes [49]. The differential effect between dsTREs and validamycin treatment may stem from their different mechanisms of action. Although RNA interference technology can reduce the expression level of TRE transcripts, due to the non-100% silencing effect and the inherent stability of the encoded enzyme, residual enzyme activity may still exist, leading to incomplete inhibition. In contrast, validamycin can directly inhibit enzyme activity by binding to the active site of the enzyme, regardless of the transcript level. The differences observed in the transcriptional regulation of Vg by dsTREs versus validamycin suggest potential variations in their reproductive regulatory mechanisms. It is noteworthy that neither treatment influenced *VgR* expression levels (Figure 1B,D), contrasting with findings reported for *S. frugiperda* [9]. This implies that TREs specifically regulate Vg synthesis without affecting its translocation into the ovary. Importantly, although *Vg* was down-regulated shortly after silencing the *TRE* gene (Figure 1B,D), egg production did not decline until later stages [49]. This may be attributed to a lag in translation relative to transcription.

Previous studies have shown that the expression of *Vg* is regulated by multiple signaling pathways, including JH, 20E, IIS, and TOR. Affecting key components of these pathways can impact embryo development and decrease hatchability in *N. lugens*, collectively forming a reproductive regulatory network [45,46,47,48]. In this study, TRE inhibitors suppressed both JH and 20E signaling pathways (Figure 2C,E,F), consistent with observations of inhibited egg development and reduced egg-laying rates, which are in agreement with prior reports [45,48]. However, dsTREs treatment did not affect the expression of *JHAMT* or *Met* (Figure 2A,B), but it upregulated the expression of *USP* and *EcR*, key receptors in the 20E signaling pathway (Figure 2C,D). This contrasts with the observed decline in fecundity [49]. Studies on *Spodoptera frugiperda* have demonstrated that downregulating cuticular 20E synthesis enzymes may be a regulated event, signaling slowed larval growth and pre-metamorphic physiological changes [36]. Additionally, host plant nutrients modulate JH and 20E levels in *S. frugiperda*, influencing migration and reproduction [3]. The alteration of the trehalose concentration in hemolymph by silencing TREs in this study may not have been sufficient to affect JH synthesis. Furthermore, since 20E upregulates TRE activity in *Antheraea pernyi* [55], we propose that the low trehalase mRNA levels observed here induced the upregulation of 20E signaling (Figure 2C) as a negative feedback mechanism to restore trehalose homeostasis.

The above analysis indicates that the effect of dsTREs on egg production was not mediated through changes in the JH and 20E levels. However, experimental results revealed that *InR2* expression was significantly downregulated after dsTREs injection (Figure 3B). Previous studies have identified two insulin-like receptors, *InR1* and *InR2*, in *N. lugens* [50,56]. Among these, *InR1* functions as a canonical insulin-like receptor activating the PI3K/Akt pathway, whereas *InR2* serves as a negative regulator of this pathway. Our experimental treatment did not significantly affect ovarian morphology, but led to reduced egg production (Figure 3E,F), findings that are consistent with the previous research [50,57]. Furthermore, dsTREs injection initially caused the downregulation of *S6K* (Figure 3D), and the RNAi-mediated downregulation of *S6K* has been shown to inhibit Vg synthesis (Figure 3H), which aligns with the trend of the *Vg* changes observed in this experiment [46]. Conversely, unlike dsTREs treatment, the injection of validamycin had no impact on the *InR2* expression levels (Figure 3F), but significantly suppressed *InR1* expression (Figure 3E). Notably, *InR1* silencing had a much stronger inhibitory effect on reproduction, which is consistent with the pronounced inhibitory effect of validamycin on *N. lugens* reproduction observed in the present study [58]. Additionally, consistent with its effects on JH and 20E, validamycin injection inhibited IIS and TOR signaling during the late stage, suggesting that there may be other pathways involved in the early-stage inhibition of reproduction via validamycin, whereas changes in the classical regulatory network of reproduction are responsible for the inhibition observed in the late stage.

Lipids, primarily in the form of triglycerides, constitute over 50% of the dry weight of the fat body and represent approximately 30–40% of the oocyte’s dry weight [59]. These findings indicate that triglycerides serve as a primary energy source for oocyte maturation and embryo development [60,61]. In oviparous insects, oocyte maturation relies not only on Vg accumulation, but also on lipid accumulation [60,61]. An interesting observation in this study was that when *N. lugens* was injected with validamycin, the abdomen exhibited abnormal enlargement compared to the control (Figure 1A), whereas the ovarian development volume was significantly smaller [49]. Given that the abdomen of female *N. lugens* is predominantly composed of ovaries and fat bodies, we hypothesized that the above phenomenon is caused by the abnormal distribution of fat bodies. Typically, the abnormal enlargement of fat bodies is closely associated with reproductive defects, potentially due to energy trade-offs—a process where resources are allocated between different physiological functions [62,63]. In contrast, abnormal lipid metabolism, a key aspect of adipocyte function, is linked to an altered fat body morphology. The blockage of triglyceride transport from the fat body to the ovary produces a phenotype consistent with that observed in this study [61,62,63]. Therefore, this study examined lipid metabolism and revealed a significant decrease in the mean expression levels of *Fas* and *AKH* following validamycin treatment (Figure 5C,D). These genes are responsible for fatty acid synthesis and lipid mobilization, respectively, and their downregulation inhibits reproduction in *N. lugens* [51,64]. However, the downregulation of *AKH* was not significant (Figure 5D), potentially due to large errors resulting from insufficient biological replicates. Correspondingly, the triglyceride content in the ovary was also significantly reduced after validamycin injection, indicating that the inhibition of TRE activity impacted lipid metabolism in the fat body, thereby reducing the amount of triglycerides transported to the ovary and ultimately leading to impaired egg development. Studies in mice have demonstrated that alginate suppresses adipocyte hypertrophy; however, the inability to utilize alginate in this study might have disrupted lipid synthesis [65].

Notably, dsTREs did not significantly affect the ovarian triglyceride content (Figure 4A,B) and ovarian morphology [49]; however, they did reduce the expression levels of *Fas* and *AKH* (Figure 5A,B). This discrepancy may be attributed to the weaker inhibitory effect of the pre-existing RNAi on TRE compared to that of validamycin.

## 5. Conclusions

In summary, both dsTREs and validamycin reduced the expression level of *Vg* without altering the level of its receptor *VgR*. However, their impacts on the reproductive regulatory network of *N. lugens* differed significantly. dsTREs did not influence the expression of *JHAMT* and *Met* within the juvenile hormone signaling pathway, but upregulated the mRNA levels of ecdysone receptor *USP* and *TOR*, while suppressing the expression of insulin-like receptors *InR2* and *S6K*. In contrast, validamycin downregulated the expression of *Met*, *USP*, *EcR*, *InR1*, *TOR* and *S6K*. Moreover, both dsTREs and validamycin inhibited *Fas* expression; however, only validamycin decreased triglyceride content in ovaries and induced abnormal abdominal enlargement of the abdomen in females.

## Figures and Tables

**Table 1:** Primers for dsRNA synthesis.

Primer Name	Accession Number	Primer Sequences (5′-3′)		Length
NlTRE1-1	FJ790319.1	F: GATGCAATCAAGGAGGTGTTATGGC	R: CGTATTCACCTCCACCTCCGT	451 bp
NlTRE1-2	KU556829.1	F: AGATGAAGGCATGTGGTTCG	R: CATCGATTCGCCAACTGGTAAGC	321 bp
NlTRE2	GQ397451.1	F: CCAACTGCTATGACACCGACAAG	R: GGGTTCAGATCCTGCCGTCGCT	440 bp
GFP	MW987535.2	F: AAGGGCGAGGAGCTGTTCACCG	R: CAGCAGGACCATGTGATCGCGC	720 bp

**Table 2:** Primers for qRT-PCR.

Primer Name	Accession Number	Primer Sequences (5′-3′)	
QNlActin	EU179847.1	F: TGGACTTCGAGCAGGAAATGG	R: ACGTCGCACTTCATGATCGAG
QNlVg	JF330416.1	F: CACTGCCCGTGCTGTGCTCTA	R: TGACTTCCTTGCTTTGCTCCC
QNlVgR	JQ040014.1	F: AGGCAGCCACACAGATAACCGC	R: AGCCGCTCGCTCCAGAACATT
QNlJHAMT	KP769805.1	F: GAACCTGCAGGCCAAACACA	R: ACCACTCGGTTGGGCTGAAT
QNlMet	KP797880.1	F: AGTGGCAGCGAGCGATGATT	R: TGAGGCGCAGCAAAAAGGAG
QNlUSP	KX431887.1	F: GGTGGAGCTGCTGAGGGAGA	R: AGCACTTGAGGCCGATGGAG
QNlEcR	FJ263049.1	F: CGAAGCCTGGAAGGTGGAGA	R: GGCAAAGATTGGCGACGATT
QNlInR1	KF974333.1	F: GAGTGCAACCCGGAGTATGT	R: TCTTGACGGCACACTTCTTG
QNlInR2	KF974334.1	F: CTCTTGCCGAACAGCCTTAC	R: GGGTCGTTTAGTGGGTCTGA
QNlTOR	JQ793898.1	F: GGCTACAGGGATGTCAAA	R: GAGATAGATTCAAACGGAAAG
QNlS6K	KP769804.1	F: AATCGGACGACTTGGAGACAGT	R: CAGTTTGGAAAGCGTACATCAGG
QNlAKH	AB817235.1	F: CCCTTCTGATGGCAGTCCTTTG	R: ATGGATGCCTTGCAGCCTTCT
QNlFas	XM_022339189.2	F: CGGAGACTCTGCCCTAA	R: CAGCGACTAATCCAACATC

## Data Availability

The original contributions presented in this study are included in the article. Further inquiries can be directed to the corresponding authors.
